# Cathepsin S in tumours, regional lymph nodes and sera of patients with lung cancer: relation to prognosis

**DOI:** 10.1054/bjoc.2001.2057

**Published:** 2001-10

**Authors:** J Kos, A Sekirnik, G Kopitar, N Cimerman, K Kayser, A Stremmer, W Fiehn, B Werle

**Affiliations:** Department of Biochemistry and Molecular Biology, Jožef Stefan Institute, Jamova 39, Ljubljana, SI-1000, Slovenia; R&D Division, Department of Biochemical Research and Drug Design, KRKA d.d., Ljubljana, SI-1000, Slovenia; Thoraxklinik, GmbH, Heidelberg, D-69126, Germany; Medizinische Klinik und Poliklinik, Ruprecht Karls University, Zentrallabor, Heidelberg, D-68115, Germany

**Keywords:** Cathepsin S, lung tumours, ELISA, monoclonal antibody, MHC-II, immunohistochemistry

## Abstract

Cysteine proteinase cathepsin S (Cat S) is expressed mainly in lymphatic tissues and has been characterised as a key enzyme in major histocompatibility complex class II (MHC-II) mediated antigen presentation. Cat S has been measured in tissue cytosols of lung parenchyma, lung tumours and lymph nodes and in sera of patients with lung tumours and of healthy controls, by specific enzyme-linked immunosorbent assay (ELISA). A difference in Cat S level was found between tumour and adjacent control tissue cytosols of 60 lung cancer patients (median 4.3 vs. 2.8 ng mg^−1^protein). In lymph nodes obtained from 24 patients of the same group, the level of Cat S was significantly higher than in tumours or lung parenchyma (*P*< 0.001). Additionally, significantly higher levels were found in non-infiltrated than in infiltrated lymph nodes (median 16.6 vs 7.5 ng mg^−1^protein). Patients with low levels of Cat S in tumours and lung parenchyma exhibited a significantly higher risk of death than those with high levels of Cat S (*P*= 0.025 – tumours;*P*= 0.02 – parenchyma). Immunohistochemical analysis (IHA) of lung parenchyma revealed a staining reaction in alveolar type II cells, macrophages and bronchial epithelial cells. In regional lymph node tissue, strong staining of Cat S was found in lymphocytes and histiocytes. Nevertheless, Cat S was detected also in tumour cells, independently of their origin. Our results provide evidence that Cat S may be involved in malignant progression. Its role, however, differs from that of the related Cats B and L and could be associated with the immune response rather than with remodelling of extracellular matrix. © 2001 Cancer Research Campaign  http://www.bjcancer.com


British Journal of Cancer (2001) 85(8), 1193–1200
© 2001 Cancer Research Campaign
doi: 10.1054/ bjoc.2001.2057, available online at http://www.idealibrary.com on IDEAL

### Cathepsin S in tumours, regional lymph nodes and sera of patients with lung cancer: relation to prognosis

**J Kos[1,2], A Sekirnik[1], G Kopitar[1], N Cimerman[2], K Kayser[3], A Stremmer[3], W Fiehn[4] and B Werle[3,4]**

[1]Jožef Stefan Institute, Department of Biochemistry and Molecular Biology, Jamova 39, SI-1000 Ljubljana, Slovenia; [2]KRKA, d.d., R&D Division, Department of Biochemical Research and Drug Design, SI-1000 Ljubljana, Slovenia; [3]Thoraxklinik, GmbH, D-69126 Heidelberg, Germany; [4]Ruprecht Karls University, Medizinische Klinik und Poliklinik, Zentrallabor, D-68115 Heidelberg, Germany

**Summary** Cysteine proteinase cathepsin S (Cat S) is expressed mainly in lymphatic tissues and has been characterised as a key enzyme in major histocompatibility complex class II (MHC-II) mediated antigen presentation. Cat S has been measured in tissue cytosols of lung parenchyma, lung tumours and lymph nodes and in sera of patients with lung tumours and of healthy controls, by specific enzyme-linked immunosorbent assay (ELISA). A difference in Cat S level was found between tumour and adjacent control tissue cytosols of 60 lung cancer patients (median 4.3 vs. 2.8 ng $mg^{-1}$ protein). In lymph nodes obtained from 24 patients of the same group, the level of Cat S was significantly higher than in tumours or lung parenchyma ($P < 0.001$). Additionally, significantly higher levels were found in non-infiltrated than in infiltrated lymph nodes (median 16.6 vs 7.5 ng $mg^{-1}$ protein). Patients with low levels of Cat S in tumours and lung parenchyma exhibited a significantly higher risk of death than those with high levels of Cat S ($P = 0.025$ – tumours; $P = 0.02$ – parenchyma). Immunohistochemical analysis (IHA) of lung parenchyma revealed a staining reaction in alveolar type II cells, macrophages and bronchial epithelial cells. In regional lymph node tissue, strong staining of Cat S was found in lymphocytes and histiocytes. Nevertheless, Cat S was detected also in tumour cells, independently of their origin. Our results provide evidence that Cat S may be involved in malignant progression. Its role, however, differs from that of the related Cats B and L and could be associated with the immune response rather than with remodelling of extracellular matrix.
© 2001 Cancer Research Campaign http://www.bjcancer.com

**Keywords**: Cathepsin S; lung tumours; ELISA; monoclonal antibody; MHC-II; immunohistochemistry

Cathepsins have been shown to participate in dissolution and remodelling of connective tissue and basement membranes in the processes of tumour growth, invasion and metastasis (Sloane et al, 1994; Kos and Lah, 1998). Increased levels of Cats B and L in tumours and some extracellular fluids are associated with shorter disease-free and overall survival periods and may serve as prognostic factors for cancer patients (Kos and Lah, 1998). The role of another cysteine proteinase, Cat S in tumour progression is less understood. In contrast to Cats B and L, which are active at acidic pH, Cat S retains most of its enzymatic activity at pH 7.0–7.5. Another distinct feature is the substantial elastolytic activity it exhibits at neutral pH (Chapman et al, 1997).

Cat S exhibits restricted tissue expression and is present in higher amounts in lymph nodes (Turenšek et al, 1975), spleen and antigen-presenting cells (APCs), including macrophages, dendritic cells and B lymphocytes (Shi et al, 1994; Morton et al, 1995). Its expression was found to be induced by cytokines, such as interferon $\gamma$ and interleukin $1\beta$ (Chapman et al, 1997).

Cat S activity appears to be essential in the MHC class II antigen presentation pathway. It specifically degrades the invariant chain (Ii), a MHC class II chaperone, prior to its removal from the MHC class II peptide-binding cleft. This facilitates the loading of antigenic peptides to MHC class II $\alpha\beta$-dimers and subsequent transportation of the complex to the cell surface to initiate MHC class II restricted $CD4^+$ T-cell recognition (Chapman et al, 1997; Pierre and Mellman, 1998). Besides participation in the immune response some other specific functions, associated with high elastolytic activity of Cat S have been proposed. For example, Cat S has been suggested to promote motility of the cilia of conducting airway cells in the lung (Chapman et al, 1997). Additionally, it was found to participate in vascular matrix remodelling and in the formation of the atherosclerotic intima (Sukhova et al, 1998). In rat, Cat S is expressed in thyroid tissue, where a role in processing thyroglobulin and release of thyroid hormone has been proposed (Petancheska and Devi, 1992). Increased expression of Cat S has also been associated with the pathogenesis of Alzheimer disease (Lemere et al, 1995).

The aim of the present study was to evaluate the role this enzyme might have in the progression of lung cancer. For this purpose a quantitative method for measuring the level of Cat S in tissue extracts and sera of lung cancer patients was developed and the levels have been tested for their relationship to clinical features, considering especially the correlation with the survival rate for cancer patients. Additionally, the cell types, expressing Cat S in tumours, lung parenchyma and lymph nodes, have been evaluated by IHA.

*Received 16 February 2001*
*Revised 19 June 2001*
*Accepted 2 July 2001*

*Correspondence to:* J Kos

### MATERIALS, METHODS AND PATIENTS

### Antigen, antibodies

Human pro-Cat S was produced in *E. coli* using an inducible T7-based expression system (Kopitar et al, 1996) and autocatalytically processed to active cathepsin S at pH 4.5 in the presence of an excess of cysteine and catalytic amounts of dextran sulfate. Active Cat S was used to immunise mice and as a standard for assay calibration curves.

Monoclonal antibodies (MAbs) (Krka, d.d., Ljubljana, Slovenia) used in ELISA, IHA and Western blots were prepared from mouse hybridoma cell lines (Schweiger et al, 1997; Zavašnik-Bergant et al, 2001). The cell culture supernatants were purified by affinity chromatography on protein A-Sepharose as recommended by the producer (Pharmacia, Uppsala, Sweden). Antibody-containing fractions were pooled, dialysed against PBS, pH 7.2, and concentrated to at least 1 mg $ml^{-1}$ by ultrafiltration. Murine MAb was conjugated to horseradish peroxidase (HRP, Type IV, Sigma, St Louis, USA) using the 2-step glutaraldehyde method (Avrameas et al, 1978).

### Western blot

Proteins were first separated by SDS-PAGE on 8–25% polyacrylamide gels (Pharmacia) using a PhastSystem apparatus and procedure (Pharmacia). After electrophoresis the proteins were transferred to a nitrocellulose membrane (Millipore, Bedford, USA) by passive diffusion. The membrane was first soaked in PBS, + 0.5% Tween 20, pH 7.2 to block non-specific binding and incubated subsequently with primary MAbs, raised against human Cat S. After washing, the membrane was incubated with 1/1000 diluted goat anti-mouse IgG conjugated to HRP (Dianova, Hamburg, Germany). Detection was performed using 0.05% 3,3′-diaminobenzidine tetrahydrochloride (DAB, Sigma) and 0.09% $H_2O_2$ in 0.05M Tris-HCl buffer, pH 7.5.

### ELISA

The combination of 2B4 MAb and 1E3 MAb (Krka, d.d., Ljubljana, Slovenia) was used to optimise sandwich ELISA. Both antibodies recognise mature and pro-forms of Cat S. Microtitre plates were coated with 10 μg $ml^{-1}$ of 2B4 MAb in 0.01 M carbonate/bicarbonate buffer, pH 9.4 at 4˚C. After blocking (2% BSA-PBS, 150 μl $well^{-1}$), the samples or Cat S standards were added (100 μl $well^{-1}$). After 2 h of incubation the wells were washed and filled with 1E3 MAb conjugated with HRP. After a further 2 h incubation at 37˚C 200 μg $well^{-1}$ of peroxidase substrate 3,3′,5,5′-tetramethylbenzidine (TMB, Sigma) 0.012% $H_2O_2$, was added. After 15 min the reaction was stopped by adding 50 μl of 2 M $H_2SO_4$. The amount of degraded substrate, as a measure of bound immunocomplexed Cat S, was determined by absorbance at 450 nm, and the concentration of Cat S calculated from the calibration curve.

### IHA of Cat S in tumours, lymph nodes and parenchyma of the lung

Sections (3–5 μm) from formalin-fixed, paraffin-embedded tissues were used for immunohistochemical analysis (Hsu et al, 1981; Kayser and Gabius, 1997). Tissue sections were deparaffinised by xylene (2 × 5 min) and rehydrated through alcohol 99% (2 × 5 min), 96% (1 × 2 min), 70% (1 × 2 min), 50% (1 × 2 min), to phosphate-buffered saline (PBS), pH 7.4 (3 × 3 min). Tissue sections were incubated with 0.1% trypsin in PBS, pH 7.4, containing 20 mM $CaCl_2$, at 37˚C for 30 min. They were then washed in distilled $H_2O$ (1 × 1 min) and equilibrated in PBS (1 × 5 min). After blocking (i) endogenous peroxidase with 3% methanolic hydrogen peroxide for 15 min, and (ii) non-specific binding sites with 5% normal goat serum in PBS for 30 min at 25˚C, tissue sections were rinsed in PBS (3 × 3 min) and incubated with a monoclonal anti-human Cat S antibody (clones 2B4 and 1E3, Krka, d.d.) diluted to 8 μg $ml^{-1}$ in PBS containing 5% normal goat serum in a humid chamber at 25˚C overnight. After washing in PBS (3 × 3 min), detection was performed by using the highly sensitive CSA-system (DAKO GmbH, Hamburg, Germany). Briefly, tissue sections were incubated with biotinylated rabbit anti-mouse Ig antibodies in 0.05 M Tris-HCl, pH 7.6 containing 5% normal goat serum. This procedure was followed by sequential 15-minute incubations with streptavidin–biotin–peroxidase complex, biotinylated thyramin–peroxidase complex and streptavidin coupled to peroxidase. After each step tissue sections were washed in PBS (2 × 5 min). Peroxidase activity was developed for 5 min with DAB (0.3 mg $ml^{-1}$ in 0.05 M Tris-HCl buffer, pH 7.6), including 0.2% hydrogen peroxide. The reaction was stopped by washing the tissue sections in distilled water for 5 min. Finally, tissue sections were lightly counterstained with 5% Harris's haematoxylin, dehydrated and mounted.

The following control assays were performed: (i) pre-adsorption of the antigen with the antibody in a 1:3 molar ratio. Cat S had been inactivated in Tris-HCl buffer, pH 8.2 at 37˚C for 30 min prior incubation with the antibody (Bromme et al, 1989), (ii) incubation as in the assay, omitting the primary antibody; (iii) incubation omitting secondary antibody, (iiii). All control assays were performed on 2 parallel sections. Tissue sections were inspected by the pathologist of the Thoraxklinik in Heidelberg, Germany (KK), classified into 2 groups and considered positive when > 5% of stained cells (tumour cells, histiocytes, lymphocytes) were present.

### Patients, samples

Matched pairs of tumour and adjacent non-cancerous (parenchyma) lung tissue were obtained from 73 patients treated by surgery at the Thoraxhospital Heidelberg-Rohrbach (Germany). 62 were primary non-small-cell lung cancer (NSCLC) tumours and 11 secondary tumours. For 24 patients from this group tumour cell infiltrated and non-infiltrated lymph nodes were also obtained. Tissue cytosols were prepared and protein concentration determined as described (Werle et al, 1995).

Serum samples were obtained from 148 patients with malignant lung tumours. Sera from 49 patients (33%) were collected before therapy whereas from the rest of the lung cancer patients ($n$ = 99) sera were collected after therapy, all in the year 1997. Out of 148 patients with malignant tumours 57 were adenocarcinoma, 57 small-cell lung cancer (SCLC) and 34 squamous cell type carcinoma. SCLC patients were treated by chemotherapy whereas non-small-cell lung carcinoma (NSCLC) patients were treated by surgery followed by chemotherapy and/or radiation therapy depending on the stage of the tumour (Manegold and Drings, 1998; Schraube et al, 1998).

A control group of sera was collected from 40 healthy donors (Kos et al, 1998), matched for gender and age to the patient's population. In all cases the blood (5 ml) was clotted at 4–8˚C, centrifuged at 3000 rpm and sera stored at –70˚C until analysed.

Tissue sections for IHA were prepared from 26 NSCLC tumours (9 adenocarcinoma, 6 squamous cell carcinoma, 6 large cell carcinoma, 5 carcinoids), 7 SCLC tumours, 3 secondary

*© 2001 Cancer Research Campaign*

tumours, 6 benign lung tissues and 4 infiltrated and 6 non-infiltrated lymph nodes.

### Statistical analysis

For descriptive statistics, SPSS PC software was used (Release 6.0, SPSS Inc, Chicago, IL). For comparing the data of matched pairs of tumour and lung tissue, Wilcoxon's rank test was applied. Differences in Cat S levels (tissue and serum) between various groups of patients and controls were tested by Mann–Whitney and Kruskal–Wallis test. Univariate analysis of survival probability was performed by Kaplan–Meier analysis (Kaplan and Meier, 1958), using the log-rank test for determining statistical significance between survival curves (Release 6.0, SPSS Inc, Chicago, IL; PC-Statistics, TOPSOFT, Hannover, Germany). Critlevel program (Abel et al, 1984) was used for separating variables into low and high groups. In all tests, 2-sided *P* values below 5% were considered significant.

### RESULTS

### Antibody specificity

MAbs used in ELISA and IHA were tested for specificity by Western blots. As evident from Figure 1, 1E3 and 2B4 MAbs reacted with the mature (28 kDa) and pro-form (36 kDa) of recombinant human Cat S. Pretreatment of both MAbs with the antigen in 1:1 to 1:3 molar ratio resulted in significant decrease of the signal. In tissue cytosols of lung tumours and lymph nodes predominantly the mature form of Cat S was detected (results not shown). MAbs did not react with the related human cathepsins B, H and L.

### Cat S protein levels in tissue cytosols and sera, determined by ELISA

A working range of 2–62 ng ml$^{-1}$ and a detection limit, defined as the concentration corresponding to the mean absorbance of 10 replicates of zero standards plus 3SDs of 0.8 ng ml$^{-1}$, were determined from the calibration curve (Figure 2). The intra-assay coefficient of variance (CV), determined by measuring 12 replicates each of low, middle and high controls, varied from 11.5% to 3.8%. Inter-assay precision, determined by evaluating 3 controls in quadruplicate in 10 separate assays, ranged from CV 7.3% to 4.3%. To test the accuracy of ELISA, different amounts of recombinant human Cat S were added to known amounts of Cat S in serum and tissue cytosol samples. Recovery levels were determined by comparing the expected vs observed concentrations and ranged from 85.2% to 116.0% (Figure 2). Additionally, tissue cytosols and sera were serially diluted to the levels encompassing the range of the assay. The linearity of the response was evaluated by comparing the measured values with the calibration values. The dilution curves for sera and tissue cytosols were linear between 1 : 4 and 1 : 60 (Figure 2). The optimal dilution of samples was defined from the linear range of dilution curves. For sera it ranged from 1/4 to 1/30, for tissue extracts from 1/6 to 1/60.

The median levels of Cat S in tumours and adjacent lung tissues of 62 NSCLC patients are shown in Table 1. The Cat S level is approximately 2 times higher in tumour than control lung tissue. No significant difference was found between adenocarcinomas and squamous cell carcinomas, whereas in secondary tumours the Cat S level was significantly lower than in primary tumours ($P$ = 0.01). Additionally, Cat S levels were not associated with tumour grade or TNM status. In non-invaded lymph node tissue obtained from the same patients the median level of Cat S was 16.6 ng mg$^{-1}$ of total protein (range 5.4–77.2) whereas in lymph nodes invaded by tumour cells, it was significantly lower-7.5 ng mg$^{-1}$ (range 2.0–84.0, $P < 0.001$). Mean values ± 2 SEM are shown in Figure 3.

In sera the median levels of Cat S were 14.2 ng ml$^{-1}$ (range 9.1–24.0) for healthy controls and 13.1 ng ml$^{-1}$ (range 8.0–28.5) for patients with malignant disease. For both groups no association with age or gender has been observed. The difference was neither

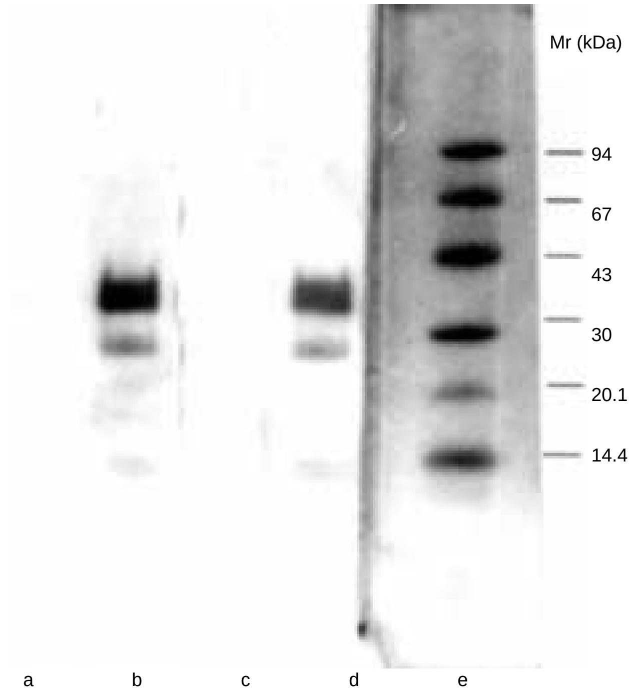

**Figure 1** Western blot of partially activated pro-Cat S developed with anti-Cat S 2B4 and 1E3 MAbs. a: developed with 2B4 MAb, pretreated with the antigen (1 : 2 molar ratio); b: as (a) but without pretreatment; c: developed with 1E3 MAb, pretreated with antigen (1 : 2 molar ratio); d: as (c) but without pretreatment; e: molecular mass standards

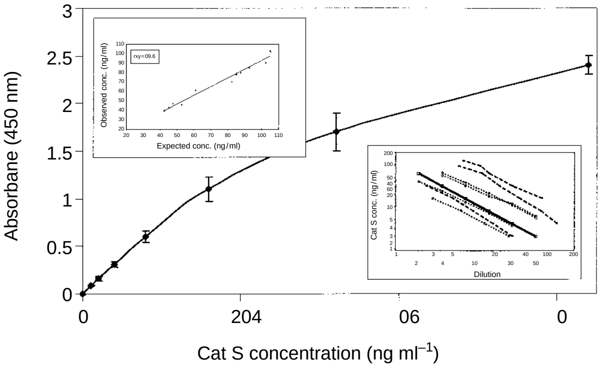

**Figure 2** Calibration curve for Cat S in ELISA. Insertion right: dilution curves of 4 serum samples (....) and 3 tissue cytosols (——) of normal lung. (—) Standard curve. Insertion left: Analytical recovery: observed vs. expected concentration of added Cat S in serum and tissue cytosol samples

© 2001 Cancer Research Campaign

**Table 1** Cat S levels in tumour and lung tissue cytosols

| | Cat S (ng mg$^{-1}$ protein) | | | |
|---|---|---|---|---|
| | ***n*** | **Tumour median (5%, 95%)** | **Lung median (5%, 95%)** | ***P* value** |
| Non-small-cell lung cancer | 62 | 4.8 (0.6, 29.2) | 2.1 (0.4, 7.6) | < 0.0001 |
| Adenocarcinomas | 31 | 4.9 (0.5, 19.8) | 1.9 (0.4, 7.7) | < 0.001 |
| Squamous cell carcinomas | 31 | 4.2 (0.6, 162) | 2.6 (0.4, 7.7) | < 0.01 |
| Secondary tumours | 11 | 3.0 (0.5, 47.6) | 0.7 (0.3, 6.2) | < 0.01 |
| Well/moderately differentiated (G1/G2) | 20 | 3.2 (0.5, 38.8) | | |
| Poorly differentiated (G3) | 57 | 4.9 (0.5, 31.7) | | |
| pTNM I | 15 | 5.0 (0.5, 29.9) | | |
| pTNM II | 11 | 4.9 (0.9, 162) | | |
| pTNM IIIa | 22 | 5.2 (0.7, 11.9) | | |
| pTNM IIIb | 12 | 3.8 (0.4, 13.2) | | |
| pTNM IV | 5 | 5.1 (1.4, 201.6) | | |

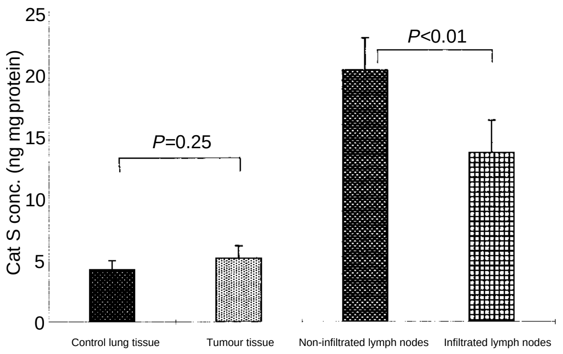

**Figure 3** Cat S levels in tumours, control lung tissue and lymph nodes of patients with lung cancer (*n* = 29). Error bars represent 2 SEM

significant between the control group and patients with malignant disease nor between groups with different histologies or between pre-therapy and post-therapy sera.

### IHA

Immunohistochemical staining of lung parenchyma, regional lymph nodes and lung tumours of different origin is shown in Figure 4. The location of Cat S in an alveolus is seen in Figure 4A, B, where a brown staining reaction is visible in alveolar type II cells and macrophages. The enzyme shows a lysosomal distribution over the cytoplasm. In addition, we observed different staining intensity of Cat S in macrophages as well as in alveolar type II cells. Immunoreactive Cat S was not found in alveolar type I cells. Furthermore, Cat S was present in a high concentration also in bronchial epithelial cells, showing either a lysosomal distribution or a more restricted localisation in the basal proliferating zone of the epithelium.

The analysis of infiltrated regional lymph node tissue revealed a strong staining of Cat S in lymphocytes whereas in non-infiltrated tissue Cat S was stained in lymphocytes and histiocytes, most likely macrophages. Cat S can also be detected in lung tumours, independently of their origin. We found a positive reaction in adenocarcinomas, squamous cell carcinomas and small cell carcinomas. Remarkably, a weak-positive reaction at the sites of interaction between tumour cells of the small cell carcinoma and the extracellular matrix of the alveoli could be observed. Correlation of Cat S staining (positive vs. negative) with clinico-pathological parameters of NSCLC patients is shown in Table 2. No significant correlation between Cat S level and grading or tumour stage has been observed. Table 3 shows IHA of Cat S in lung tumours, secondary tumours, benign lung tissue and infiltrated and non-infiltrated lymph nodes with regard to staining intensity in tumour cells, histiocytes and lymphocytes.

### Survival analysis

Cat S levels in tumour tissue cytosols and adjacent control lung parenchyma from 60 patients with NSCLC were included in univariate survival analysis. Median follow-up of the observed tumour patients was 3.0 years (25th percentile: 1.09 years). Cut-off values of 5.0 ng mg$^{-1}$ for tumour tissue and 0.5 ng mg$^{-1}$ for lung tissue have been optimised using the Critlevel program (Abel et al, 1984). In both groups, patients with the levels above the cut-off values experienced significantly better survival probability than the patients with lower Cat S levels ($P$ = 0.025 – tumours;

 *© 2001 Cancer Research Campaign*

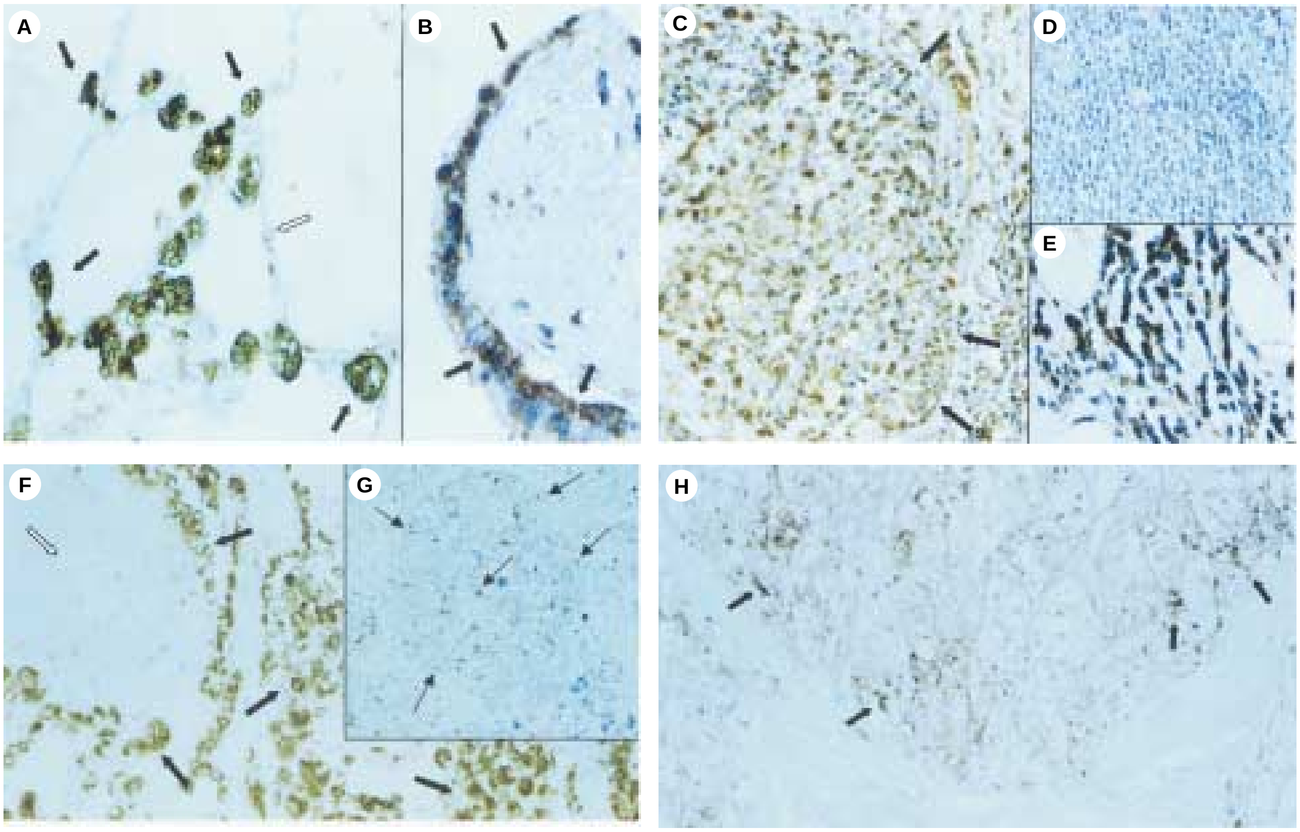

**Figure 4** IHA of Cat S in lung parenchyma. (**A**) An alveolus consisting of alveolar type I cells, alveolar type II cells and macrophages. Alveolar type I cells did not stain (white arrow), while alveolar type II cells and macrophages show a granular cytoplasmic staining pattern (black arrow). (**B**) Bronchial epithelial cells show a lysosomal distribution for Cat S and a positive reaction in the basal proliferating zone of the epithelium as well (black arrow). In regional lymph nodes, (**C**) and (**E**) show the typical sinusoid structure of a lymph node containing different types of lymphocytes (black arrow). Lymphocytes and histiocytes, most probably macrophages, stained strongly positive. (**D**) Shows the corresponding negative control. **F**–**H** show lung tumours of different histologies. (**F**) Adenocarcinoma located around a blood vessel. The cytoplasm of tumour cells stained positive (black arrow). Erythrocytes and connective tissue clearly did not stain (white arrow). (**G**) A weak positive reaction could be observed at sites of interaction between tumour cells of a small cell carcinoma and the extracellular matrix of the alveoli, but also within the tumour (small black arrows). Lymphocytes did not stain. (**H**) Squamous cell carcinoma surrounded with connective tissue. Tumour cells show a faint positive reaction (black arrows)

$P = 0.02$ – parenchyma) (Figure 5). In addition, survival curves are significantly separated in the first 2 years of follow-up.

### DISCUSSION

Lysosomal cysteine proteinases are known to mediate intracellular protein turnover, regulating the lifetime of proteins critical for normal cell function. They are also involved in specific processing steps for smaller peptides, pro-hormones and pro-enzymes (Uchiyama et al, 1994). Some cysteine proteinases such as Cats B, L and H, are expressed in almost all cells, suggesting a housekeeping function in protein turnover, whereas the expression of others, e.g. Cats S, K and V is restricted to particular tissues and cells, indicating more specific roles in cell physiology.

Cat S is distinguished from the other members of papain superfamily by its high stability at neutral pH and expression, restricted to lymphatic tissues and antigen-presenting cells (Wiederanders et al, 1991; Brömme et al, 1994). Some of the specific properties are consistent with subtle structural differences found in the active site of Cat S by crystal structure analysis (McGrath et al, 1998; Gunčar et al, 1999). Due to the localisation in lymphatic tissues and cells it was suggested that Cat S is involved in modulating the immune response (Chapman et al, 1997; Kirschke et al, 1998). Its specific role in processing the invariant chain (Ii) designates this enzyme as a potentially useful therapeutic target in diseases associated with a hyperimmune response, such as asthma, hypersensitive pneumonitis and autoimmune disorders (Chapman et al, 1997). Since there is an increasing body of evidence that impaired antigen-processing might also be associated with malignant transformation and escape of tumour cells from recognition by T cells (Seliger et al, 2000), the knowledge on tumour Cat S could help to characterise the molecular mechanisms regulating presentation of tumour-associated antigens (TAA).

On the other hand, Cat S expresses broad endopeptidase activity at neutral pH, so one may expect it to play a role in extracellular tissue remodelling associated with tumour invasion and metastasis. Indeed, in our previous study we found that the increased activity of Cat S is associated with lung tumours (Werle et al, 1999). Quantitative determination of the protein levels of Cat S should provide additional insight into the role of this enzyme in malignant processes. For this reason we have developed a quantitative ELISA, using 2 monoclonal antibodies recognising distinct epitopes on Cat S molecule. Both MAbs, 1E3 and 2B4, used in the assay recognize the pro-form (36 kDa) and the mature form (28

© 2001 Cancer Research Campaign 

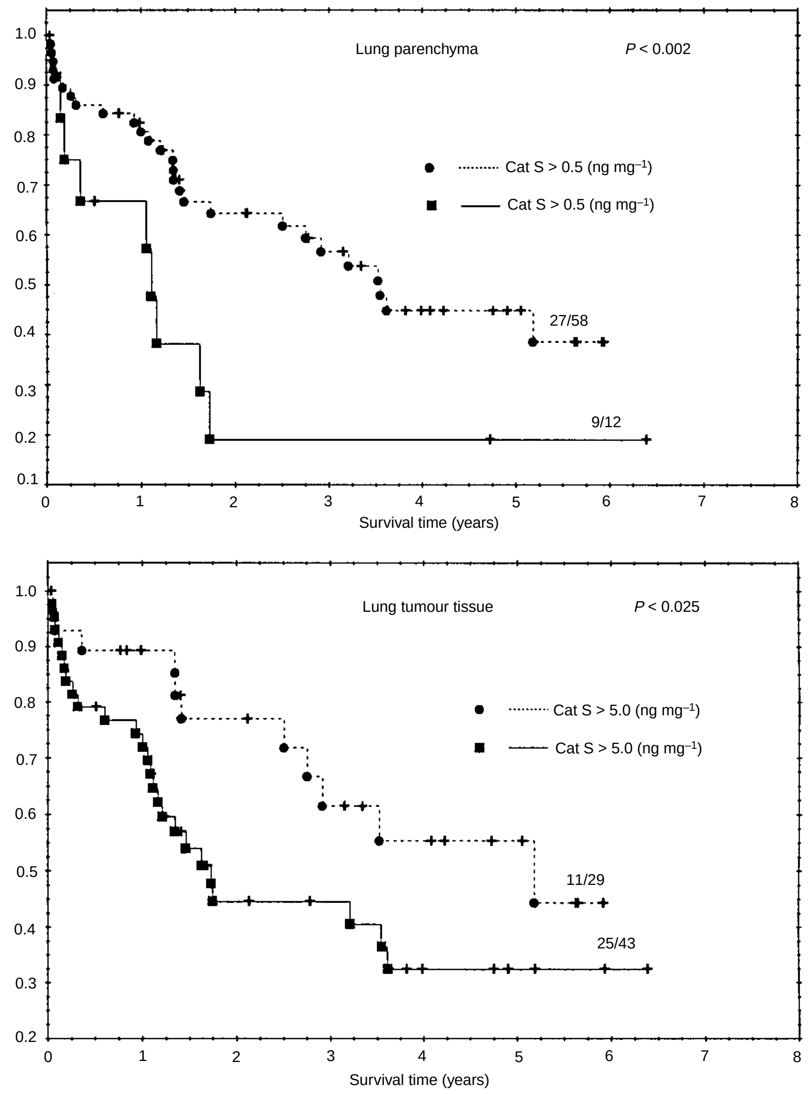

**Figure 5** Prognostic significance of Cat S levels in parenchyma (**A**) and tumours (**B**) of lung cancer patients. Patients with Cat S levels below cut-off value had a significantly shorter overall survival (■ deceased patients, uncensored data; + still living patients, censored data) than patients with Cat S levels above cut-off value (● deceased patients, uncensored data;+ still living patients, censored data)

**Table 3** Immunohistochemical analysis of Cat S in lung tumours and infiltrated lymph nodes

| | | **Tumour cells** | | **Histiocytes** | | **Lymphocytes** | |
|---|---|---|---|---|---|---|---|
| | ***n*** | **yes** | **no** | **yes** | **no** | **yes** | **no** |
| Malignant lung tissue | 36 | | | | | | |
| Non-small-cell lung cancer | 26 | 16 | 10 | 7 | 19 | 6 | 20 |
| Adenocarcinomas | 9 | 6 | 3 | 2 | 7 | 1 | 8 |
| Squamous cell carcinomas | 6 | 2 | 4 | 1 | 5 | 1 | 5 |
| Large cell carcinoma | 6 | 4 | 2 | 2 | 4 | 2 | 4 |
| Carcinoid | 5 | 4 | 1 | 2 | 3 | 2 | 3 |
| Small cell lung cancer | 7 | 5 | 2 | 1 | 6 | 2 | 5 |
| Secondary tumours[a] | 3 | 1 | 2 | 1 | 2 | 0 | 3 |
| Benign lung tissue[b] | 6 | | | 3 | 3 | 1 | 5 |
| Lymph node tissue | | | | | | | |
| Infiltrated | 4 | 2 | 2 | 0 | 4 | 2 | 2 |
| Non-infiltrated | 6 | | | 2 | 4 | 2 | 4 |

[a]Tumours of other organs to the lung.
[b]Focal reaction in alveolar duct cells.

 *© 2001 Cancer Research Campaign*

**Table 2** Correlation of Cat S staining in tumour tissue sections with clinico-pathological parameters

| | *n* | Negative | Positive |
|---|---|---|---|
| G1/2 | 8 | 5 | 3 |
| G3 | 18 | 9 | 9 |
| pT1 | 3 | 1 | 2 |
| pT2 | 16 | 10 | 6 |
| pT3 | 4 | 3 | 1 |
| pT4 | 3 | 2 | 1 |
| pN0 | 15 | 10 | 5 |
| pN1 | 4 | 2 | 2 |
| pN3 | 0 | 0 | 0 |
| pTNM I | 8 | 5 | 3 |
| pTNM II | 2 | 1 | 1 |
| pTNM IIIa | 9 | 6 | 3 |
| pTNM IIIb | 1 | 1 | 0 |
| pTNM IV | 6 | 3 | 3 |

Tissue sections were considered positive when more than 5% of cells were stained. The tumour disease stage was classified according to the international staging system (Hermanek and Sobin, 1987).

kDa) of recombinant and natural antigen as shown by Western blot analysis. The new ELISA enables reliable and accurate quantification of Cat S in tissue cytosols and sera of lung cancer patients.

The levels of Cat S found in lung tissue and lymph nodes agree well with previous reports on the higher expression of this enzyme in lymphatic tissues. Its mean level was 5 times higher in lymph nodes than in lung parenchyma. The levels of Cat S in tumour tissues were found to be higher than those in control parenchyma from the same patients, revealing a possible role for this enzyme in tumour growth and progression. However, serum levels of Cat S were not significantly changed from control levels in patients with malignant disease. Although in our previous report we showed also an increased enzymatic activity of Cat S in lung tumours (Werle et al, 1999), it seems that Cat S is not involved in tumour development and progression to the same extent as are the related cysteine proteinases Cats B and L. These are significantly over-expressed in lung tumours, released to the extracellular space and participate in degradation of extracellular matrix proteins (Kos and Lah, 1998). The lack of any correlation of Cat S activity or protein levels with clinical and pathological parameters indicating tumour progression, is consistent with the latter conclusion. In IHA, a positive immunostaining reaction in tumour tissue was observed not only in tumour cells but also in epithelial cells, histiocytes and infiltrated lymphocytes, which may all significantly contribute to the higher overall Cat S protein content and activity. In normal lung tissue a significant staining reaction was observed in alveolar type II cells and macrophages and in bronchial epithelial cells. Its localisation in the basal proliferating zone of the epithelium is consistent with the proposed role of this enzyme in the motility of cilia of conducting lung airway cells (Chapman et al, 1997).

Cat S protein levels in infiltrated lymph nodes revealed a significant decrease compared with non-infiltrated lymph nodes. By contrast, the Cat B level was the highest in infiltrated lymph nodes, as shown in our previous study (Werle et al, 2000). From IHA it is evident that Cat S staining in regional lymph nodes is the most extensive in lymphocytes and histiocytes whereas Cat B was predominantly present in tumour cells, partially in histiocytes and absent in lymphocytes. Lower levels of Cat S could be related to the lower expression in affected lymphoid tissue or to the lower number of lymphatic cells. A tumour-directed immune response usually results in enlargement of lymph nodes as a consequence of the increase of the number of lymphoid cells (Lores et al, 1998). However, this vigorous process is limited to the early growth of tumours and is soon down-regulated, permitting progressive tumour growth and proliferation (Barna and Deodhar, 1975).

There is growing evidence that $CD4^+$ T cells play a central role in initiating and effecting antitumour immunity. TAA can be presented to $CD4^+$ T cells by professional APCs cells as well as by MHC class $II^+$ tumour cells. For direct antigen presentation these tumour cells must leave primary tumour site and enter the lymphatic system and draining lymph nodes. The expression of MHC class II molecules in tumour cells varies among different tumour types and has been associated with both, poor and good prognosis of cancer patients (Seliger et al, 2000). The expression of other proteins involved in class II antigen presentation and their relation to the course of disease have not been systematically analysed. We have no direct evidence that Cat S is involved in regulation of antitumour immunity, however, we may speculate that its high expression in potential APCs could be beneficial for cancer patients. The latter is consistent with the survival analysis of Cat S in lung cancer patients. It confirmed different pattern for Cat S compared with other cathepsins. Numerous clinical studies on different cancer types have provided evidence that patients with high levels of Cats B and L show higher risk of relapse or death (Kos et al, 1998). In contrast, in this study patients with high levels of Cat S experienced significantly better survival probability than those with low levels of Cat S. Prognostic value was defined not only for tumour samples but, even more significantly, for control lung parenchyma. The latter further indicates that increased expression of Cat S is not linked just to the tumour cells and their proliferation and invasion but merely reflects defence mechanisms leading to the regression of malignant disease and better outcome of cancer patients.

In conclusion, the results of our study reveal an increased expression of Cat S in lung tumours. Its role, however, does not seem to be pivotal in remodelling extracellular matrix proteins, resulting in tumour growth, proliferation and metastasis. Its high expression in lymphocytes and histiocytes, decreased levels in infiltrated lymph nodes and a correlation of low levels with poor patient survival, could be related to the modulation of antigen presentation and consequently the response to tumour antigens in cancer patients.

### ACKNOWLEDGEMENTS

This work was supported by grants J3-6208 and RA 2320 (JK) from the Ministry of Science and Technology of the Republic of Slovenia, by a grant of the Deutsche Krebshilfe – Dr Mildred Scheel-Stiftung (10-1985-Eb 1) and in part by the Zentrallabor of the Medizinische Klinik and Poliklinik of the University Heidelberg. The authors thank Mrs Marjana Šenk for excellent technical assistance and Prof Roger Pain for critical reading of the manuscript. Furthermore, we thank Prof Werner Ebert and Prof Vito Turk for continuous support and encouragement of this work, Dr Thomas Muley for computer supported imaging of tissue sections, Clemens Kraft for collecting lymph node tissues, Beate Schaufler for excellent handling of patients' data and Dr Werner Rittgen for his help in statistical evaluation.

*© 2001 Cancer Research Campaign*

### REFERENCES

Abel U, Berger J and Wiebelt H (1984) Critlevel. An exploratory procedure for the evaluation of quantitative prognostic factors. *Methods Inf Med* **23**: 154–156

Avrameas S, Ternynck T and Guedson JL (1978) Coupling of enzymes to antibodies and antigens. *Scand J Immunol* **8**: 7–23

Barna B and Deodhar SD (1975) The activity of regional nodes in the evolution of immune response to alogenetic and geneic tumors. *Cancer Res* **35**: 920–926

Brömme D, Steinert A, Friebe S, Fittkau S, Wiederanders B and Kirschke H (1989) The specificity of bovine spleen cathepsin S: a comparison with rat liver cathepsins L and B. *Biochem J* **264**: 475–481

Brömme D, Bonneau PR, Lachance P and Storer AC (1994) Engineering the S2 subsite specificity of human cathepsin S to a cathepsin L and cathepsin B-like specificity. *J Biol Chem* **269**: 30238–30242

Chapman HA, Riese JR and Shi GP (1997) Emerging roles for cysteine proteinases in human biology. *Ann Rev Physiol* **59**: 63–88

Gunčar G, Pungerčič G, Klemenčič I, Turk V and Turk D (1999) Crystal structure of MHC class II-associated p41 Ii fragment bound to cathepsin L reveals the structural basis for differentiation between cathepsins L and S. *EMBO J* **18**: 793–803

Hermanek P and Sobin L (1987) UICC TNM classification of malignant tumors, 4th edn. Springer Verlag: Berlin

Hsu SM, Raine L and Fanger H (1981) Use of avidin-biotin peroxidase complex (ABC) in immunoperoxidase techniques: a comparison between ABC and unlabeled antibody (PAP) procedures. *J Histochem Cytochem* **29**: 577–580

Kaplan EL and Meier P (1958) Nonparametric estimation from incomplete observation. *J Am Stat Assoc* **53**: 457–481

Kayser K and Gabius HJ (1997) Graph theory and the entropy concept in histochemistry. Theoretical considerations, application in histopathology and the combination with receptor-specific approaches. In: *Progress in Histochemistry and Cytochemistry*, Graumann W, Bendayan M, Bosman FT, Heitz PU, Larsson LI, Wolfe HJ, eds. Vol 32, Gustav Fisher: Sttutgart, Jena, Lubeck, Ulm

Kirschke H, Wideranders B, Brömme D and Rinne A (1998) Cathepsin S from bovine spleen: Purification, distribution, intracellular localization, and action on proteins. *Biochem J* **264**: 467–473

Kopitar G, Dolinar M, Štrukelj B, Pungerčar J and Turk V (1996) Folding and activation of human procathepsin S from inclusion bodies produced in *Escherichia coli*. *Eur J Biochem* **236**: 558–562

Kos J and Lah TT (1998) Cysteine proteinases and their endogenous inhibitors: Target proteins for prognosis, diagnosis and therapy in cancer (Review). *Oncology Reports* **5**: 1349–1361

Kos J, Nielsen HJ, Krašovec M, Christensen IJ, Cimerman N, Stephens RW and Brünner N (1998) Prognostic values of cathepsin B and carcinoembryonic antigen in sera of patients with colorectal cancer. *Clin Cancer Res* **4**: 1511–1516

Lemere CA, Munger JS, Shi GP, Natkin L, Haass C, Chapman HA and Selkoe DJ (1995) The lysosomal cysteine protease, cathepsin S, is increased in Alzheimer's disease and Down syndrome brain. An immunohistochemical study. *Am J Pathol* **146**: 848–860

Lores B, Garcia-Estevez JM and Arias C (1998) Lymph nodes and human tumors (review). *Int J Mol Med* **1**: 729–733

Manegold C and Drings P (1998) Chemoterapie des nichtkleinzelligen Lungenkarzinoms. In: *Thoraxtumoren: Diagnostik-Staging-gegenwartiges Terapiekonzept*, Drings P, Vogt-Moykopf I, eds. pp. 310–327, Springer Verlag, Heidelberg

McGrath ME, Palmer JT, Brömme D and Somoza JR (1998) Crystal structure of human cathepsin S. *Protein Science* **7**: 1294–1302

Morton PA, Zacheis ML, Giacoletto KS, Manning JA and Schwartz BD (1995) Delivery of nascent MHC class II invariant chain complexes to lysosomal compartments and proteolysis of invariant chain of cysteine proteases precedes peptide binding in B-lymphoblastoidic cells. *J Immunol* **154**: 137–150

Petancheska S and Devi L (1992) Sequence analysis, tissue distribution, and expression of rat cathepsin S. *J Biol Chem* **267**: 26038–26043

Pierre P and Mellman I (1998) Developmental regulation of invariant chain proteolysis controls MHC class II trafficking in mouse dendritic cells. *Cell* **93**: 1135–1145

Schraube P, Kimmig B, Latz D, Flentje M and Wannenmacher M (1998) Radiotherapie des Bronchialkatzinoms. In: *Thoraxtumoren: Diagnostik-Staging-gegenwartiges Terapiekonzept*, Drings P, Vogt-Moykopf I, eds. pp. 277–295, Springer Verlag, Heidelberg

Schweiger A, Štabuc B, Popovič T, Turk V and Kos J (1997) Enzyme-linked immunosorbent assay for the detection of total cathepsin H in human tissue cytosols and sera. *J Immunol Methods* **201**: 165–172

Seliger B, Maeurer MJ and Ferrone S (2000) Antigen-processing machinery breakdown and tumor growth. *Immunology Today* **21**: 455–464

Shi GP, Webb AC, Foster KE, Knoll JHM and Lemere CA (1994) Human cathepsin S: chromosomal localisation, gene structure, and tissue distribution. *J Biol Chem* **269**: 11530–11536

Sloane BF, Moin K and Lah TT (1994) Lysosomal enzymes and their endogenous inhibitors in neoplasia. In: *Biochemical and Molecular Aspects of selected Cancers*, Pretlow TG and T.P. Pretlow TP eds. Pp 411–466, Academic Press, New York

Sukhova GK, Shi GP, Simon DI, Chapman HA and Libby P (1998) Expression of the elastolytic cathepsins S and K in human atheroma and regulation of their production in smooth muscle cells. *J Clin Invest* **102**: 576–583

Turnšek T, Kregar I and Lebez D (1975) Acid sulphydril protease from calf lymph-nodes. *Biochim Biophys Acta* **403**: 514–520

Uchiyama Y, Waguri S, Sato N, Watanabe T, Ishido K and Kominami E (1994) Review: Cell and tissue distribution of lysosomal cysteine proteinases, cathepsins B, H and L, and their biological roles. *Acta Histochem Cytochem* **27**: 287–308

Werle B, Ebert W, Klein W and Spiess E (1995) Assessment of cathepsin L activity by use of the inhibitor CA-074 compared to cathepsin B activity in human lung tumor tissue. *Biol Chem Hoppe-Seyler* **376**: 157–164

Werle B, Staib A, Julke B, Ebert W, Zlatoidsky P, Sekirnik A, Kos J and Spiess E (1999) Fluorimetric microassays for the determination of cathepsin L and cathepsin S activities in tissue extracts. *Biol Chem* **380**: 1109–1116

Werle B, Kraft C, Schanzenbacher U, Lotterle H, Kayser K, Lah T, Kos J, Ebert W and Spiess E (2000) Significant association of cathepsin B and the lymphatic metastasising process. *Cancer* **89**: 2282–2291

Wiederanders B, Bromme D, Kirschke H, Kalkkiner N and Rinne A (1991) Primary structure of bovine cathepsin S. Comparison to cathepsins L, H and B. *FEBS Lett* **286**: 189–192

Zavašnik-Bergant T, Sekirnik A, Golouh R, Turk V and Kos J (2001) Immunochemical localisation of Cathepsin S, Cathepsin L and MHC Class II-Associated p41 isoform of invariant chain in human lymph node tissue. *Biol Chem* **380**: 799–804

*© 2001 Cancer Research Campaign*

## References

[PDF_00688] Abel U., Berger J., Wiebelt H. (1984). CRITLEVEL: an exploratory procedure for the evaluation of quantitative prognostic factors.. Methods Inf Med.

[PDF_00692] Barna B., Deodhar S. D. (1975). The activity of regional nodes in the evolution of immune responses to allogeneic and isogeneic tumors.. Cancer Res.

[PDF_00697] Brömme D., Bonneau P. R., Lachance P., Storer A. C. (1994). Engineering the S2 subsite specificity of human cathepsin S to a cathepsin L- and cathepsin B-like specificity.. J Biol Chem.

[PDF_00694] Brömme D., Steinert A., Friebe S., Fittkau S., Wiederanders B., Kirschke H. (1989). The specificity of bovine spleen cathepsin S. A comparison with rat liver cathepsins L and B.. Biochem J.

[PDF_00700] Chapman H. A., Riese R. J., Shi G. P. (1997). Emerging roles for cysteine proteases in human biology.. Annu Rev Physiol.

[PDF_00702] Guncar G., Pungercic G., Klemencic I., Turk V., Turk D. (1999). Crystal structure of MHC class II-associated p41 Ii fragment bound to cathepsin L reveals the structural basis for differentiation between cathepsins L and S.. EMBO J.

[PDF_00708] Hsu S. M., Raine L., Fanger H. (1981). Use of avidin-biotin-peroxidase complex (ABC) in immunoperoxidase techniques: a comparison between ABC and unlabeled antibody (PAP) procedures.. J Histochem Cytochem.

[PDF_00719] Kirschke H., Wiederanders B., Brömme D., Rinne A. (1989). Cathepsin S from bovine spleen. Purification, distribution, intracellular localization and action on proteins.. Biochem J.

[PDF_00722] Kopitar G., Dolinar M., Strukelj B., Pungercar J., Turk V. (1996). Folding and activation of human procathepsin S from inclusion bodies produced in Escherichia coli.. Eur J Biochem.

[PDF_00725] Kos J., Lah T. T. (1998). Cysteine proteinases and their endogenous inhibitors: target proteins for prognosis, diagnosis and therapy in cancer (review).. Oncol Rep.

[PDF_00728] Kos J., Nielsen H. J., Krasovec M., Christensen I. J., Cimerman N., Stephens R. W., Brünner N. (1998). Prognostic values of cathepsin B and carcinoembryonic antigen in sera of patients with colorectal cancer.. Clin Cancer Res.

[PDF_00731] Lemere C. A., Munger J. S., Shi G. P., Natkin L., Haass C., Chapman H. A., Selkoe D. J. (1995). The lysosomal cysteine protease, cathepsin S, is increased in Alzheimer's disease and Down syndrome brain. An immunocytochemical study.. Am J Pathol.

[PDF_00735] Lores B., García-Estevez J. M., Arias C. (1998). Lymph nodes and human tumors (review).. Int J Mol Med.

[PDF_00740] McGrath M. E., Palmer J. T., Brömme D., Somoza J. R. (1998). Crystal structure of human cathepsin S.. Protein Sci.

[PDF_00743] Morton P. A., Zacheis M. L., Giacoletto K. S., Manning J. A., Schwartz B. D. (1995). Delivery of nascent MHC class II-invariant chain complexes to lysosomal compartments and proteolysis of invariant chain by cysteine proteases precedes peptide binding in B-lymphoblastoid cells.. J Immunol.

[PDF_00748] Petanceska S., Devi L. (1992). Sequence analysis, tissue distribution, and expression of rat cathepsin S.. J Biol Chem.

[PDF_00750] Pierre P., Mellman I. (1998). Developmental regulation of invariant chain proteolysis controls MHC class II trafficking in mouse dendritic cells.. Cell.

[PDF_00757] Schweiger A., Stabuc B., Popovíc T., Turk V., Kos J. (1997). Enzyme-linked immunosorbent assay for the detection of total cathepsin H in human tissue cytosols and sera.. J Immunol Methods.

[PDF_00760] Seliger B., Maeurer M. J., Ferrone S. (2000). Antigen-processing machinery breakdown and tumor growth.. Immunol Today.

[PDF_00762] Shi G. P., Webb A. C., Foster K. E., Knoll J. H., Lemere C. A., Munger J. S., Chapman H. A. (1994). Human cathepsin S: chromosomal localization, gene structure, and tissue distribution.. J Biol Chem.

[PDF_00769] Sukhova G. K., Shi G. P., Simon D. I., Chapman H. A., Libby P. (1998). Expression of the elastolytic cathepsins S and K in human atheroma and regulation of their production in smooth muscle cells.. J Clin Invest.

[PDF_00772] Turnsek T., Kregar I., Lebez D. (1975). Acid sulphydryl protease from calf lymph nodes.. Biochim Biophys Acta.

[PDF_00778] Werle B., Ebert W., Klein W., Spiess E. (1995). Assessment of cathepsin L activity by use of the inhibitor CA-074 compared to cathepsin B activity in human lung tumor tissue.. Biol Chem Hoppe Seyler.

[PDF_00784] Werle B., Kraft C., Lah T. T., Kos J., Schanzenbächer U., Kayser K., Ebert W., Spiess E. (2000). Cathepsin B in infiltrated lymph nodes is of prognostic significance for patients with nonsmall cell lung carcinoma.. Cancer.

[PDF_00781] Werle B., Staib A., Jülke B., Ebert W., Zladoidsky P., Sekirnik A., Kos J., Spiess E. (1999). Fluorometric microassays for the determination of cathepsin L and cathepsin S activities in tissue extracts.. Biol Chem.

[PDF_00787] Wiederanders B., Broemme D., Kirschke H., Kalkkinen N., Rinne A., Paquette T., Toothman P. (1991). Primary structure of bovine cathepsin S. Comparison to cathepsins L, H, B and papain.. FEBS Lett.

[PDF_00790] Zavasnik-Bergant V., Sekirnik A., Golouh R., Turk V., Kos J. (2001). Immunochemical localisation of cathepsin S, cathepsin L and MHC class II-associated p41 isoform of invariant chain in human lymph node tissue.. Biol Chem.

